# Decentralizing testing capacity of mpox in Africa: a narrative review

**DOI:** 10.1016/j.lanafr.2026.100046

**Published:** 2026-07

**Authors:** Yao Selom Atrah, Nicksy Gumede, Joseph Nyombe, Nebiyu Dereje, Wazih N. Cho, Kyeng Mercy, Michel Muteba, Jeanine Nkakulu Luzolo, Olga Ntumba-Tshitenge, Passy Kimema, Collins Kipngetich Tanui, Noah Takah Fongwen, Armel Mbouna, Rose Mary Nakame, Biruh Tesfaye Kebede, Yap Boum, Ngashi Ngongo, Mory Keita, Sofonias Kifle Tessema, Yenew Kebede Tebeje, Jean Kaseya

**Affiliations:** a Africa Centres for Disease Control and Prevention (Africa CDC), Addis Ababa, Ethiopia; b Mpox Continental Incident Management Support Team (IMST), Kinshasa, Democratic Republic of the Congo; c World Health Organization, African Region, Brazzaville, Democratic Republic of the Congo; d Soins de Santé Primaires en Milieu Rural (SANRU), Kinshasa, Democratic Republic of the Congo

**Keywords:** Mpox, Testing, Decentralization, Africa, Outbreak, Laboratory, Primary care

## Abstract

Decentralized diagnostics are essential for the timely detection and control of mpox outbreaks. Burundi and the Democratic Republic of Congo (DRC) transitioned from centralized testing to decentralized models using GeneXpert platforms, mobile labs, and real-time feedback systems. Burundi expanded from one to 56 diagnostic sites, reducing site-level turnaround times to 2–4 h and achieving nearly 100% testing coverage. The DRC scaled up from 2 to 28 labs, deploying multiplex platforms and stabilizing testing rates. Genomic surveillance also advanced, with over 3000 mpox virus (MPXV) genomes sequenced across 23 countries, revealing new clades and informing public health responses. Africa reached the 8% sequencing threshold, enabling real-time viral tracking. The mpox epidemic accelerated reforms, including continental guidelines for laboratory decentralization and the launch of a new initiative to expand testing, training, and local manufacturing. This article describes the mpox laboratory decentralization efforts, achievements, lessons, and best practices across Africa.

**Research in context**

**Evidence before this study**

We reviewed the existing literature on mpox testing and response via Google Scholar, PubMed, and Web of Science as of Oct 25, 2025, using the search terms mpox OR monkeypox AND testing OR laboratory, with no date or language restrictions. We have also reviewed available literature from national, regional and World Health Organization (WHO) reports. Mpox has been endemic in Africa since the 1970s, yet surveillance and laboratory testing have remained suboptimal, contributing to under-detection and delayed response. During the 2022–2023 global outbreak affecting over 116 countries, Africa accounted for less than 16% of laboratory-confirmed cases despite being heavily affected. Limited diagnostic capacity, exemplified by the Democratic Republic of Congo, where testing was centralized in two national laboratories serving over 112 million people, resulted in historically low confirmation rates—only 18% of suspected cases were tested in 2024.

**Added value of this study**

This article demonstrates the transformative impact of decentralizing mpox diagnostics in Africa, shifting from reliance on centralized national laboratories to a network of regional and district-level facilities equipped with GeneXpert platforms and mobile labs. By implementing a phased decentralization strategy in Burundi and the DRC, site-level turnaround times for test results dropped from 7 to 10 days to just 2–4 h, enabling timely case confirmation, improved surveillance, and rapid outbreak control. The approach also integrated innovative diagnostic algorithms, optimized resource use, and strengthened cross-border preparedness. These findings provide a scalable model for enhancing diagnostic equity and resilience in resource-limited settings, offering lessons for future epidemic responses across the continent.

**Implications of all the available evidence**

The evidence underscores that centralized diagnostic systems are insufficient for timely epidemic control in Africa, particularly for diseases like mpox that often emerge in remote areas. Decentralization of laboratory capacity, combined with innovative diagnostic algorithms and integrated surveillance, significantly improves outbreak detection, reduces turnaround times, and enhances response effectiveness. These findings highlight the need for sustained investment in decentralized, flexible diagnostic networks and point-of-care technologies as part of Africa's health security agenda. Such systems should be institutionalized to strengthen preparedness for future epidemics and other public health threats.

## Introduction

Early warnings of emerging risks from localized outbreaks in Africa are often overlooked, leading to a gap in surveillance, laboratory testing, and timely response. Mpox is one example of such a neglected disease on the continent.^1^ Since its initial detection in Africa in the 1970s, mpox has persistently affected several countries across the continent. In 2022–2023, the disease spread globally, affecting more than 116 countries and territories.^2^ Despite Africa being among the most affected continents, it contributed to a relatively low proportion (<16%) of the global laboratory-confirmed cases during this period. While sub-optimal surveillance played a role in these lower reported numbers, inadequate laboratory capacity was a significant contributing factor to under-detection. For instance, the Democratic Republic of Congo (DRC), which contributed significantly to the continental mpox case burden in 2022, had notably limited testing capacity. Laboratory confirmation was largely centralized in two national reference laboratories run by the Institut National de la Recherche Biomédicale (INRB) in Kinshasa and Goma, serving a population of over 112 million inhabitants, and historically, less than one-third of reported cases have been laboratory confirmed; for example, only about 18% were tested in 2024.^3^ The limitations of centralized diagnostic models during outbreaks are well documented, with multiple studies demonstrating that decentralizing molecular diagnostics improves access to testing, shortens site level turnaround time, and strengthens outbreak control.^4^, ^5^, ^6^, ^7^

On August 13, 2024, the Africa Centres for Disease Control and Prevention (Africa CDC) declared the mpox outbreak a Public Health Emergency of Continental Security (PHECS). This alert was quickly followed by a declaration of a Public Health Emergency of International Concern (PHEIC) by the WHO the next day. This declaration led to the development of a continental mpox preparedness and response plan, followed by the revised mpox continental response plan 2.0. Both plans emphasized intensified surveillance, integration of diagnostics into existing laboratory platforms, coordinated interventions and the implementation of sustainable measures to strengthen preparedness for future epidemics.^8^ At the continental level, Africa CDC and WHO played key roles in coordinating the response through the incident management support team (IMST), which brought together more than 28 partners under the 4-One principle: One Team, One Plan, One Budget, and One Monitoring and Evaluation Framework. Within this coordination mechanism, the IMST established key laboratory performance indicators to guide implementation and monitoring across Member States. These included: (i) testing coverage, defined as the proportion of suspected cases tested (target ≥80%); (ii) testing rate, referring to the percentage of samples tested out of the total number of samples received by the laboratory (target 100%); (iii) laboratory turnaround time, calculated as the interval between sample collection and the release of validated test results (target median ≤3 days); and (iv) sequencing rate, defined as the proportion of confirmed cases that were sequenced (target ≥5%). Through this mechanism, Member States received guidance aligned with the International Health Regulations (IHR 2005) to support their national mpox responses.^9^

Between July 2024 and July 2025, a total of 163,399 suspected cases were reported across 28 African Union Member States. Of these, 100,375 samples (61.4%) were collected and tested, with 42,270 laboratory-confirmed positive cases (42.1% positivity rate), including 226 deaths, resulting in a case-fatality rate of 0.5%. However, the true burden of the disease in Africa remains uncertain due to limited surveillance and testing capacity. The DRC emerged as the epicenter of the outbreak, accounting for 58% of confirmed cases and 49% of reported deaths, prompting urgent calls for a coordinated response strategy.^10^

Laboratories play a critical role in the mpox response by enabling case confirmation, guiding clinical care, and informing surveillance and control measures. In addition, genomic sequencing is essential to monitor the evolution of the virus and to adjust the response, ensuring timely adaptation of strategies at both national and continental levels. However, reliance on centralized laboratories could limit the response capacity, including delays in case confirmation, resulting in postponed patient management and vaccination, high sample transport costs, and restricted access for remote communities. To address these challenges, we have implemented a decentralization of mpox laboratory services in DRC and Burundi. Here, we describe the crucial role of decentralizing laboratory diagnostic capacity and intensifying genomic surveillance in Africa's response to mpox. Drawing on concrete case studies from Burundi and the DRC, it highlights the successes, challenges, and lessons learned from the rapid expansion of laboratory capacity under emergency conditions. In this context, the mpox epidemic offers Africa the opportunity to “turn the tide” on diagnostic inequities and establish sustainable laboratory networks as a legacy that can serve as a model for preparing for future outbreaks.

## Methods

A structured review of the literature on mpox diagnostics and laboratory response was conducted using Google Scholar, PubMed, and Web of Science up to Oct 25, 2025, with the search terms “mpox” OR “monkeypox” AND “testing” OR “laboratory”, without date or language restrictions. We additionally reviewed relevant national, regional, and WHO reports. Operational data from the mpox continental IMST were derived from national situation reports submitted weekly by Member States and consolidated into a joint Africa CDC–WHO database through the IMST Surveillance Pillar, notably via the Analytics Unit. These data, covering the period July 2024 to August 2025, were analyzed descriptively to assess trends in diagnostic capacity expansion, laboratory turnaround time, testing coverage, and genomic sequencing output.

Burundi and the DRC were purposively selected as illustrative case studies based on their epidemiological relevance and contrasting health system contexts. According to the WHO Regional Office for Africa mpox regional bulletin of 8 December 2024,^11^ the Democratic Republic of the Congo reported the highest number of laboratory-confirmed mpox cases in the African Region in 2024 (11,984 cases), followed by Burundi (2523 cases). While the DRC illustrates laboratory decentralization in a large, conflict-affected setting with major logistical and security constraints, Burundi demonstrates the feasibility of rapid nationwide decentralization in a smaller, lower-resource system under emergency conditions, together offering complementary and transferable lessons on scalability, feasibility, and system-wide coverage.

Laboratory performance indicators were defined using the IMST framework: testing coverage (proportion of suspected cases tested), testing rate (proportion of received samples tested), laboratory turnaround time (interval between sample collection and release of validated results), and sequencing rate (proportion of confirmed cases sequenced). Analyses were descriptive, and observed trends were interpreted alongside concurrent investments in surveillance, logistics, workforce deployment, and partner support, without attributing causality solely to laboratory decentralization. This approach was selected to support policy-oriented analysis of laboratory system strengthening under emergency conditions rather than a systematic review or causal effectiveness assessment.

## Key findings

### The crucial role of decentralized diagnostics

Without the ability to accurately and rapidly detect cases, outbreaks can spread silently, undermining control efforts. For mpox in Africa, diagnostics have historically been centralized in National Reference Laboratories, usually located in capital cities or major urban centers. While this model is effective for quality assurance, it is deeply dependent on a well-funded supply and logistics chain, including comprehensively tracked sample transportation, and has shown limitations in supporting timely case detection in rural and hard-to-reach areas where mpox is often endemic.^12^

The 2022–2024 mpox epidemic exposed the fragility of these centralized systems. Countries like Burundi, Uganda, and the DRC experienced significant delays in case confirmation, with turnaround times for test results ranging from 5 to 14 days (up to 90 days have been observed in some provinces of the DRC) during the early phases of the outbreak.^13^ These delays hindered contact tracing, slowed case management, limited vaccination, and allowed community transmission to continue unchecked. The lack of decentralized testing also placed enormous pressure on logistics systems, amid dwindling funding for last-mile sample transportation, as samples needed to be transported over long distances, often through regions with poor infrastructure or security concerns.^14^

MPXV testing methods primarily rely on molecular diagnostics, with nucleic acid amplification tests (NAATs), such as PCR, being the gold standard. These methods detect MPXV virus DNA in patient samples, such as lesion swabs, with high sensitivity and specificity. A major challenge is the absence of Antigenic Rapid Test (AgRDT) testing tools. AgRDT, as Point-Of-Care (PoC) testing, would allow for diagnostics to be performed near patients, reducing delays caused by sample transportation to centralized laboratories.^15^ The lack of rapid, reliable PoC diagnostics impedes timely detection and isolation of cases, particularly in remote regions of the DRC, where centralized laboratories are often inaccessible. The outbreak also highlighted the critical need for multiplex testing platforms. Diseases like mpox, measles, and varicella (chickenpox) often co-circulate and present with similar clinical symptoms, leading to misdiagnosis.^16^

### Laboratory decentralization lessons from Burundi and the DRC

The challenges of mpox testing were particularly pronounced in countries with the highest mpox burden, including Burundi and the DRC. The lack of laboratory capacity and testing delays significantly hampered the response to the epidemics. In response to these challenges, Africa CDC, WHO, and other partners worked to decentralize the diagnostic infrastructure.

The decentralization strategy was designed as a phased, risk-based approach to rapidly reduce diagnostic turnaround time while preserving test quality, biosafety, and data integrity. Rather than creating parallel emergency laboratory systems, the response deliberately leveraged existing national laboratory networks and molecular platforms already deployed at peripheral levels, particularly GeneXpert. Expansion was prioritized in high-burden and high-risk areas and aligned with site readiness, availability of trained personnel, and logistical feasibility. Importantly, human capacity strengthening, supervision, and quality assurance were embedded within the decentralization process to ensure both rapid scale-up and sustainability beyond the emergency phase.

#### Burundi

At the onset of its outbreak in July 2024, Burundi relied exclusively on the National Reference Laboratory in Bujumbura for mpox diagnostics. This centralized approach created significant bottlenecks, with site-level turnaround times averaging 7–10 days, delaying case confirmation and hindering effective outbreak control. In response, the Ministry of Health, with support from Africa CDC, WHO, and partners, designed and implemented a phased laboratory capacity decentralization strategy that was closely aligned with epidemic severity, site readiness, and existing laboratory minimal capacity requirements. These included geographical accessibility, trained personnel, functional and reliable equipment, continuous power supply, and the availability of reagents and consumables.

In the absence of any mpox rapid diagnostic test approved to date by Africa CDC or WHO, Burundi adopted a tiered diagnostic approach leveraging existing capacities. Decentralization was initiated through peripheral screening using the existing GeneXpert (Cepheid, Sunnyvale, CA, USA) machine network, enabling rapid testing close to communities. Where capacity existed, clade-specific confirmation was performed using qPCR-based testing at regional laboratories, while genomic sequencing remained centralized at the national reference laboratory, supported by structured sample referral pathways. This tiered approach enabled timely case confirmation while maintaining the capacity to distinguish circulating clades and monitor viral evolution. Rather than creating parallel emergency systems, the strategy intentionally leveraged existing laboratory infrastructure and personnel, including GeneXpert platforms already deployed for tuberculosis and HIV programs, as well as mobile laboratory deployment, thereby strengthening and integrating mpox diagnostics within the national laboratory network. As a result, the decentralization was extended to regional laboratories across 15 additional districts, each representing a province with limited capacity. This extension also targeted migration corridors and cross-border regions, including Muyinga, Ngozi, and Isale, that border Tanzania, Rwanda and DRC considered epidemiologically high risk. Finally, the decentralization reached peripheral districts such as Matana, Vumbi and Busoni without confirmed cases but considered as high risk.

Personnel training was embedded within the decentralization strategy: a cascade training model was adopted, with national trainers supporting regional and district teams through targeted training on mpox molecular testing, biosafety and biosecurity, sample handling, and data reporting. Refresher training, supportive supervision, and routine performance monitoring were maintained throughout implementation. Monitoring and evaluation were integrated throughout the implementation process, with key performance indicators including turnaround time, positivity rate, number of tests performed, coverage by district, and laboratory functionality. Data were consolidated into weekly reports and into DHIS2-supported dashboards.

In addition to the decentralization of testing across 51 laboratories in all health districts, intensified surveillance systems and strengthened community engagement contributed to a sharp increase in reported suspected cases, from a weekly average of 36 before August 2024 to 217 between August 2024 and May 2025. Testing rate was maintained at 100%, reflecting complete testing of received samples, while testing coverage remained close to 100% and above the IMST benchmark of 80% for suspected cases tested. The positivity rate increased from 38% before August 2024 to 51% by the end of 2024, then decreased to 47% by the end of May 2025, reflecting both improved surveillance sensitivity and expanded access to diagnostics.

**Fig. 1:**
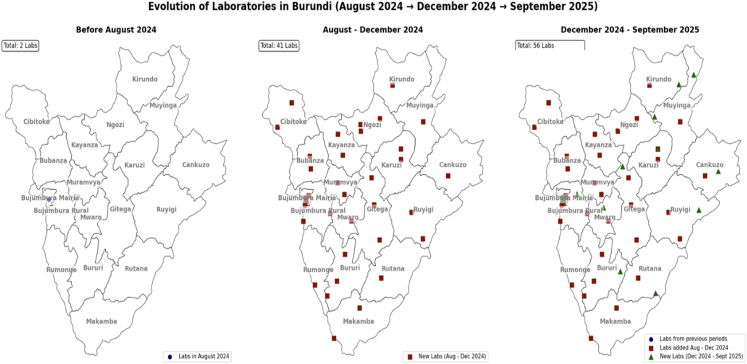
Evolution of mpox Testing Laboratories in Burundi (August 2024 → December 2024 → September 2025).

Site-level turnaround times dropped substantially from over 7–10 days before decentralization to an average of just 2–4 h once mobile laboratories and GeneXpert-based onsite testing were deployed and fully operational. By May 2025, the number of functional diagnostic sites had expanded from a single central laboratory to 56 GeneXpert-equipped facilities nationwide, enabling timely, localized responses across nearly all health districts (Fig. 1).

The decentralization of mpox diagnostics in Burundi was associated with improved outbreak response capacity. Despite a more than sixfold increase in suspected cases, the system maintained full testing coverage, while diagnostic delays were reduced to less than 48 h in most districts. The increase in positivity rates from 38% before August 2024 to 51% by the end of the year was consistent with strengthened case definition practices and intensified active case finding, which enabled the detection of more true cases. The subsequent decline to 47% by May 2025 illustrates both the broader availability of diagnostic services, which enabled testing of milder or non-mpox cases, and a gradual reduction in community transmission. Most importantly, this intervention showed that even a fragile health system, when supported by strong partner coordination and guided by a phased and well-funded decentralization strategy, can rapidly expand diagnostic capacity and establish a resilient, sustainable network able to respond effectively to public health emergencies.^13^ Building on this success, the experience directly informed the development of Burundi's national laboratory decentralization strategy, institutionalizing outbreak lessons into long-term health system strengthening.

#### Democratic Republic of the Congo

Initially limited to just two laboratories in Kinshasa and Goma, the DRC began scaling up its mpox diagnostic infrastructure in response to surging case numbers. Similar to Burundi, mpox diagnostic decentralization in the DRC followed a tiered laboratory network model adapted to a larger, conflict-affected setting. The training, supervision, and workforce strategies described above were applied to the DRC context, with additional emphasis on supporting operations in security- and logistics-constrained environments. Initial screening was decentralized to provincial and sub-national laboratories using GeneXpert platforms to reduce turnaround time, while confirmatory qPCR testing, clade differentiation, and genomic sequencing were conducted at INRB Kinshasa and Goma. By September 2024, diagnostic services had been decentralized to 8 laboratories across 8 provinces, out of the 26 that make up the territory, with a total surface area of 2,345,410 km^2^. Despite this progress, testing coverage remained low at around 39%, with turnaround times exceeding 90 days in some health zones. These delays severely hindered case confirmation and placed considerable strain on the central system.

By December 2024, the network had expanded to 12 laboratories strategically distributed across provinces, which brought the testing rate to 100%, yet testing coverage remained at 35%, as surveillance improved and new challenges in sample transportation emerged. Significant challenges persisted, with less than one-third of suspected cases being sampled, fewer than 40% of samples reaching the laboratories within 48 h, and turnaround times remaining unacceptably long. The situation deteriorated further towards the end of the first quarter of 2025, as the ongoing armed conflict in the eastern provinces disrupted health services and the transport of samples. This was compounded by the withdrawal of major United States government funding that had previously supported logistics for specimen transport. These setbacks were associated with a marked decline in testing coverage, which fell below 15%, leaving many suspected cases unconfirmed and delaying outbreak control efforts.

**Fig. 2:**
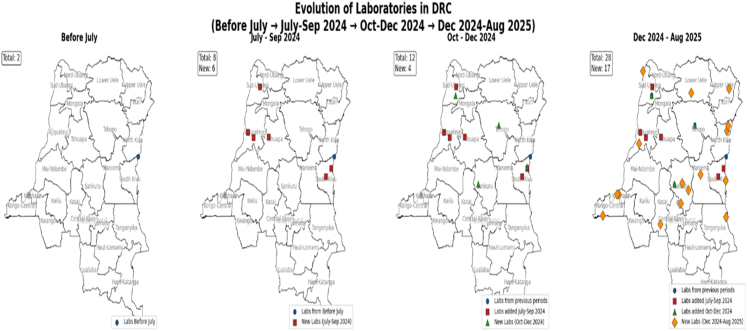
Evolution of mpox Testing Laboratories in DRC (Before July 2024 → July–September 2024→ October–December 2024 → December 2024–August 2025).

In response, a coordinated surge was launched under the “intensification” phase of the continental mpox response plan 2.0, reinforcing investments, technical assistance, and the deployment of community health workers and epidemiologists to support surveillance, case investigation, sample collection, and transport, enabling the decentralization process to regain momentum. As a result, the number of operational mpox laboratories for mpox detection rose from 12 to 23 in the second quarter of 2025 and ultimately reached 28 by August 2025 (Fig. 2), representing one of the fastest diagnostic scale-up efforts on the continent.

By mid-2025, the testing rate had stabilized at close to 100%, with approximately 90% of specimens tested within their respective provinces. These efforts translated into measurable improvements in diagnostic performance: testing coverage rebounded sharply, ranging from <30% to 76% over a period of 6 weeks (Epi week 20-Epi week 25), reaching a peak of 86% during epidemiological week 21 of 2025. Furthermore, over 70% of samples were delivered to laboratories within 48 h of collection, and more than 80% of results were returned to clinicians within 48 h, a remarkable progress compared to the delays observed earlier.

By August 2025, six Radi One (KH Medical, Seoul, South Korea) multiplex platforms had been deployed across six provinces (Kinshasa, Kasai, Sankuru, Tanganyika, Tshuapa, and Tshopo) to enable real-time detection of mpox, measles, and chickenpox, as part of the IMST integration strategy. By December 2025, with World Bank support, the Radi One network is expected to expand to 66 laboratories, covering all 26 provinces of the DRC and integrating cholera testing. In parallel, one Standard™ M10 (SD Biosensor, Suwon, Republic of Korea) point-of-care molecular diagnostic device was installed in Bukavu district (South Kivu) to complement the existing molecular diagnostic capacity, which includes 34 GeneXpert machines and 10 qPCR platforms, of which four are currently functional.

Despite these gains, significant challenges remain, particularly in sustaining a continuous supply of reagents and consumables, harmonizing quality-control systems across decentralized sites, and ensuring the long-term sustainability of operations in fragile health system settings. These developments highlight the dual reality of the DRC response: while decentralization has strengthened system resilience and improved the timeliness of epidemic control, sustaining these advances will require continued investment in supply chains, external quality assurance, equipment maintenance, and community engagement to secure lasting impact.^17^^,^^18^ These lessons demonstrate the feasibility of rapid scale-up of laboratory capacity under emergency conditions, while also underscoring the need for long-term investments to sustain diagnostic capacity beyond the crisis.

### Genomic surveillance: a pillar of preparedness

Between January 2024 and August 2025, over 3000 complete or near-complete MPXV genomes were generated from clinical samples collected across 23 African Union Member States, based on data shared with Africa CDC by national reference laboratories. This achievement was made possible through the support of Africa CDC, particularly via the Africa Pathogen Genomics Initiative (Africa PGI), and other international partners. Africa CDC provided critical resources, including sequencing reagents, equipment, and capacity building in bioinformatics analysis and interpretation, enabling affected Member States to rapidly scale up genomic surveillance. This effort, spanning West, Central, East, Northern, and Southern Africa, marks a transformative milestone in pathogen genomics, offering the most comprehensive view to date of MPXV clade diversity and evolution on the continent.

Maintaining real-time genomic surveillance is substantially more resource-intensive than routine molecular diagnostics. While PCR-based mpox diagnostics were rapidly decentralized to sub-national laboratories to support epidemic control, genomic sequencing largely remained a centralized or regionally hub-based function, reflecting requirements for infrastructure, biosafety, bioinformatics, and workforce. This approach reflects a tiered laboratory network that links decentralized molecular screening with centralized or regional sequencing via structured sample referral pathways. Despite facing severe limitations, including shortages of reagents, trained personnel, and uneven geographic coverage, African laboratories successfully identified the B.1 lineage linked to the international outbreak and documented the emergence of new clades, such as Clade Ib in the DRC.^19^^,^^20^ These genomic insights refined our understanding of viral evolution and transmission dynamics, informed timely public health responses, and underscored the strategic value of integrating genomics into epidemic preparedness.

**Fig. 3:**
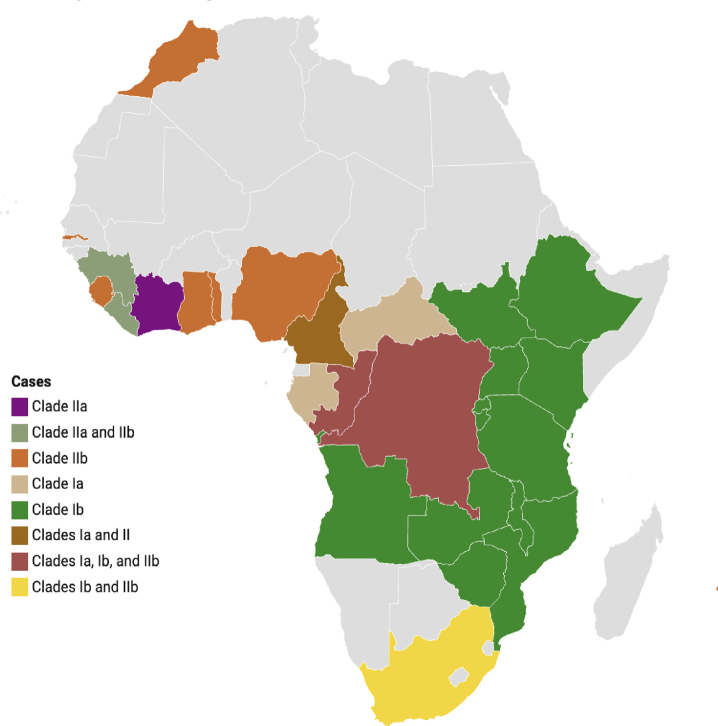
Geographical distribution of MPXV clades (Clade I and II) in Africa from January 2024 to August 2025.

The impact of genomics has led to a better understanding of MPXV dynamics and its clades. Sequencing has highlighted different modes of transmission across clades, variations in virulence, and other drivers that have guided countries in contextualizing their responses. The multiple outbreaks caused by Clades Ia, Ib, IIa, and IIb demonstrated the disease's diversity and reinforced the adaptability of the response—an achievement that would not have been possible without genomic evidence (Fig. 3).

The continent reached an estimated 8% sequencing rate of confirmed mpox cases (n = 42,270), exceeding the ≥5% benchmark defined in the continental response plan for epidemiologically and geographically representative sequencing. This capacity led to the detection of Clade IIb in the Republic of the Congo and the Democratic Republic of Congo, where previously only Clade I viruses had been reported. Similarly, it enabled the identification of the B.1 lineage (Clade IIb) in Sierra Leone,^21^ where MPXV sequencing had only recently started and is now being extended to other pathogens such as HIV and HPV.

Genomic data were primarily used for population-level outbreak monitoring and strategic response adaptation rather than for individual case management. Although sequencing turnaround times varied across countries, genomic data were monitored weekly at country level and consolidated into monthly analyses at IMST level to inform strategic and operational decision-making. While near–real-time sequencing remains the optimal standard, this structured reporting mechanism ensured that genomic insights were generated within epidemiologically meaningful timeframes for public health action.

The mpox epidemic has catalyzed a paradigm shift in Africa's approach to genomic surveillance, revealing both the continent's scientific resilience and the urgent need for sustainable infrastructure. By combining thousands of newly generated genomes with epidemiological data, public health institutes aim to construct a pan-continental genomic atlas that illuminates cross-border transmission pathways, emerging clades, and zoonotic spillover events.^22^ The generated dataset provides a robust framework for outbreak prediction, diagnostic refinement, vaccine development, and tailored control strategies. A key lesson from the mpox response is that sequencing must be embedded into routine public health systems, not treated as an ad-hoc research activity. Africa's experience offers a replicable model for other regions seeking to build equitable, decentralized genomic surveillance networks capable of responding to future health threats.

### The legacy of MPXV laboratory decentralization: sustainable preparedness

The mpox outbreak clearly demonstrated how neglected outbreaks can quickly become global health threats. It is neither the first nor the last virus to emerge and spread worldwide.^23^ Yet, the challenges of the epidemic created a unique opportunity to reform African health systems and establish a lasting legacy for continental health security.

One of the most important lessons is the recognition that decentralization is not merely a crisis measure, but a structural necessity for resilient health systems. Previous experiences, such as the massive scale-up of molecular platforms and point-of-care testing during the COVID-19 response, demonstrated both the potential and the challenges of decentralization. However, limited efforts to sustain those capacities after the emergency revealed critical gaps. Similarly, reliance on a handful of central reference laboratories during the mpox outbreak created severe bottlenecks, leading to diagnostic delays and a high toll in human lives and health.^24^

Observed improvements coincided with multiple reinforcing interventions, including enhanced surveillance, logistics, community engagement, and partner support. Outcomes cannot be attributed solely to laboratory decentralization. Evidence from previous Ebola and tuberculosis responses indicates that reduced diagnostic turnaround time and expanded testing coverage are associated with earlier case isolation, improved contact tracing, and more timely targeting of public health interventions.^4^^,^^7^ In response, IMST, in collaboration with Member States and technical partners, initiated the drafting of the Continental Guidelines for Laboratory Decentralization in Epidemic Preparedness and Response. These guidelines aim to institutionalize lessons from mpox while drawing on COVID-19, positioning decentralization as a long-term investment in health security. They emphasize the need to sustain and integrate the laboratory capacities expanded during outbreaks, grounding recommendations in the practical experiences of countries such as Burundi and the DRC. The guidelines rest on three principles: bringing diagnostics closer to communities by leveraging existing platforms such as GeneXpert; adopting phased, adaptive decentralization strategies; and integrating diagnostics with a One Health approach and genomic surveillance, recognizing the zoonotic nature of mpox.

From a sustainability perspective, maintaining skills and staff motivation beyond the emergency phase remains a key challenge. Integrating decentralized mpox testing sites within existing national laboratory networks, rather than creating parallel emergency structures, offers a pragmatic pathway to sustain training through routine supervision, refresher courses, and integration into national laboratory career frameworks. However, long-term sustainability will depend on predictable financing and continued investment in laboratory workforce development.

Building on this momentum, Africa CDC and the European Commission launched in June 2025 the partnership to accelerate mpox testing and sequencing in Africa (PAMTA), a landmark initiative co-funded under the EU4Health 2024 Work Program and implemented jointly with the African Society for Laboratory Medicine (ASLM). PAMTA represents the first formal Africa–European Union partnership on diagnostics and was conceived as a direct continuation of the mpox response legacy. It aims to deliver over 150,000 mpox tests, strengthen genomic sequencing capacity across the continent, build human resource expertise in molecular diagnostics, genomics, and bioinformatics, and promote the local manufacturing and validation of diagnostic kits within Africa. This will accelerate the transition of mpox response in national health systems.

By embedding decentralization within continental guidelines and coupling it with initiatives such as PAMTA, Africa is not only consolidating the hard-won gains of the mpox response but also laying a robust foundation for pandemic preparedness. This dual focus on bringing diagnostics closer to people while investing in genomics and manufacturing capacity marks a decisive step towards resilient, agile, and self-reliant public health systems that can withstand future epidemic threats.

## Key lessons learned and challenges

Observed improvements in diagnostic performance and response indicators occurred alongside concurrent investments in surveillance, workforce deployment, logistics, community engagement, and partner support; therefore, causal attribution to laboratory decentralization alone should be interpreted with caution. Key lessons from recent health security responses in Africa underscore the importance of systemic transformation. Decentralization has proven effective, reducing diagnostic delays, saving lives, and building public trust by bringing services closer to communities. Strategic partnerships, among continental institutions, national governments, and global partners, have significantly amplified impact, demonstrating that coordinated action is more powerful than isolated efforts. The pandemic also highlighted that genomic surveillance is no longer optional; it must be embedded into routine public health systems to anticipate and respond to emerging viral threats.

However, the response also revealed persistent gaps, particularly in One Health integration, the failure to effectively coordinate human, animal, and environmental health systems remains a critical weakness that must be addressed to prevent future zoonotic outbreaks. Funding fragility remains a critical concern, as heavy reliance on donor financing leaves laboratory systems vulnerable to collapse once global attention shifts. Data-sharing gaps between Member States and continental institutions hinder timely and coordinated responses, weakening regional surveillance and preparedness. Workforce retention is another pressing issue, with many skilled professionals leaving due to inadequate incentives and career development opportunities.

Additionally, Africa's dependence on imported reagents and sequencing platforms exposes the continent to global supply chain disruptions, while reliance on foreign technicians for equipment maintenance delays preventive and corrective servicing, limiting the autonomy of national laboratories. Finally, domestic investment in laboratory systems remains uneven, with some governments making commendable efforts, while others continue to underfund critical infrastructure, jeopardizing the sustainability of gains made through external support.

## Conclusion

The mpox outbreak has exposed Africa's vulnerabilities but has also catalyzed innovations, partnerships, and frameworks that, if sustained, could fundamentally transform the continent's epidemic preparedness. The path forward is clear: accelerate decentralization, strengthen genomic surveillance, integrate One Health, build local manufacturing, and institutionalize continental coordination.

## Contributors

YSA, NG, JN, ND, MM, YB, NN, MK, SKF, YKT, and JK conceptualized the manuscript. YSA, WNC, KM, and ND drafted the original draft of the manuscript. MM, JNL, ONT, PK, CKT, NTF, AM, RMN, BTK, YB, NN, MK, SKT, YKT, and JK validated the data and provided critical input on the manuscript. YSA has access to the data used in this manuscript. All authors have approved the final version of this manuscript for submission for publication.

## Editor note

The Lancet Group takes a neutral position regarding territorial boundaries in published maps and institutional affiliations.

## Declaration of interests

The authors declare no competing interests.
